# Supplementary material for: Systemic Dysfunction of Osteoblast Differentiation in Adipose-Derived Stem Cells from Patients with Multiple Myeloma
Source: Cells. 2019 May 10;8(5):441. doi: 10.3390/cells8050441 (PMC6562713; doi:10.3390/cells8050441)
Supplement: Supplementary file 1 [file cells-08-00441-s001.pdf]

cells

MDPI

Sup Table 1. Characteristics of the patients

**Variables**		**Patients (N=11)**
Median age, years	Median (range)	68 (50-84)
Patient sex, n (%)	Male	6 (55)
	Female	5 (45)
SD staging, n (%)	I	3 (27)
	II	7 (64)
	III	1 (9)
ISS staging, n (%)	I	8 (73)
	II	1 (9)
	III	2 (18)
Type of myeloma protein, n (%)	Ig G	11 (100)
	Ig A	0 (0)
	light chain	0 (0)
Bone marrow aspirate: % plasmacytosis	Median (range)	27 (6-80)
Bone lesions		
No lesion		3 (27%)
Minor lesions (≤3 lesions)		2 (18%)
Major lesions (>3 lesions)		5 (45%)
Not assessed		1(9%)

Abbreviations: SD: Salmon and Durie. ISS: International Staging System.

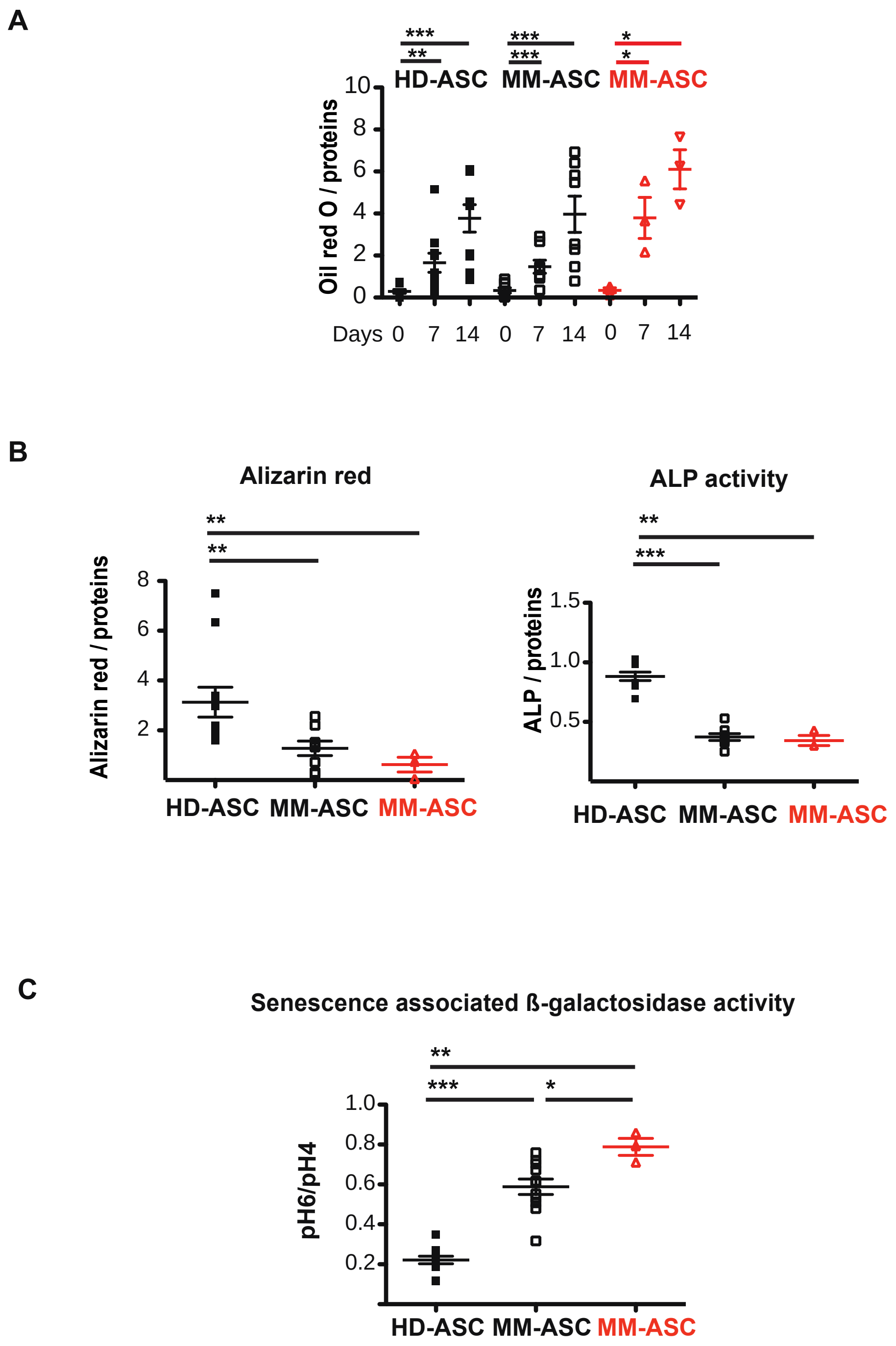

Figure Sup 1: Incidence of bone lesions status on MM-ASC alterations: A HD- and MM-ASC with (empties blacks squares) and without (red triangles) bone lesion were differentiated into adipocytes for 7 or 14 days. The cells were stained with Oil-red-O to visualize lipid droplets. Staining was quantified at 520 nm and normalized to the protein content (right). **B** ASC were differentiated into osteoblasts for 14 days. The cells were stained with Alizarin Red to visualize calcium deposition. Staining was quantified at 560 nm and normalized to the protein content (Left). Alkaline phosphatase (ALP) activity was measured (right). **C** SA β-galactosidase activity was assessed according to the ratio of pH 6- to pH 4-positive staining after 14 days of osteoblast differentiation. * $p < 0.05$, ** $p < 0.01$, *** $p < 0.001$.

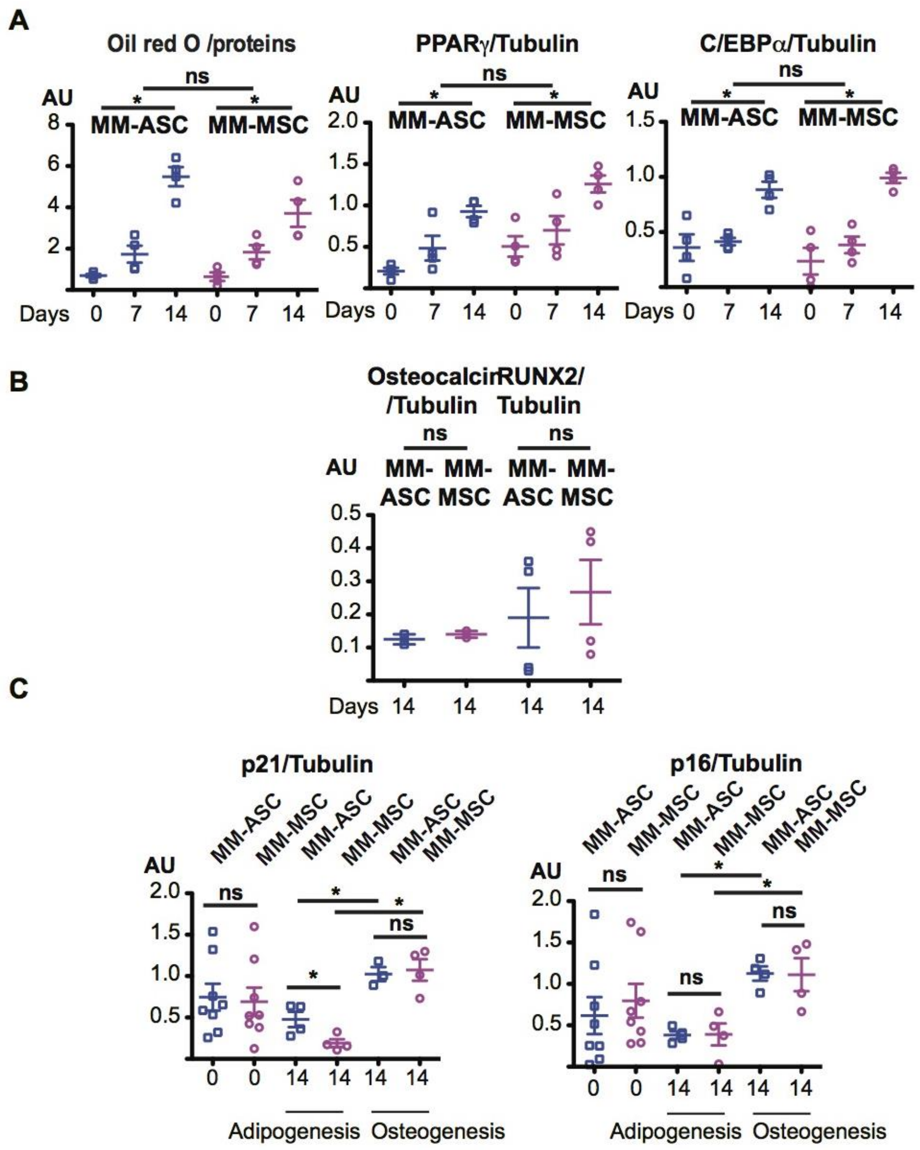

Figure Sup 2: Comparison of MM-ASC and MM-MSC within the same patients: A Both MM-ASC (in blue) and MM-MSC (in pink) were differentiated into adipocytes for 7 and 14 days. Cells were stained with Oil-Red-O to visualize lipid droplets. Staining was quantified at 520 nm and normalized to protein content (left panel). Whole cell lysates were extracted at day 0, 7 or 14 of differentiated MM-ASC (in blue) and MM-MSC (in pink) and analyzed by immunoblotting. Tubulin was used as loading control. Western blot quantifications are shown (right panel). * $p < 0.05$ *vs*. D0- ns not significant. **B** Both MM-ASC (in blue) and MM-MSC (in pink) were differentiated into osteoblasts for 14 days. Whole cell lysates were extracted and analyzed by immunoblotting. Western blot quantifications are shown. ns not significant **C** Both MM-ASC (in blue) and MM-MSC (in pink) were differentiated into adipocytes or osteoblasts for 14 days as indicated. Whole cell lysates were extracted and analyzed by immunoblotting. Western blot quantifications are shown. *$p < 0.05$ Adipocyte *vs*. Osteoblast- ns sot significant.

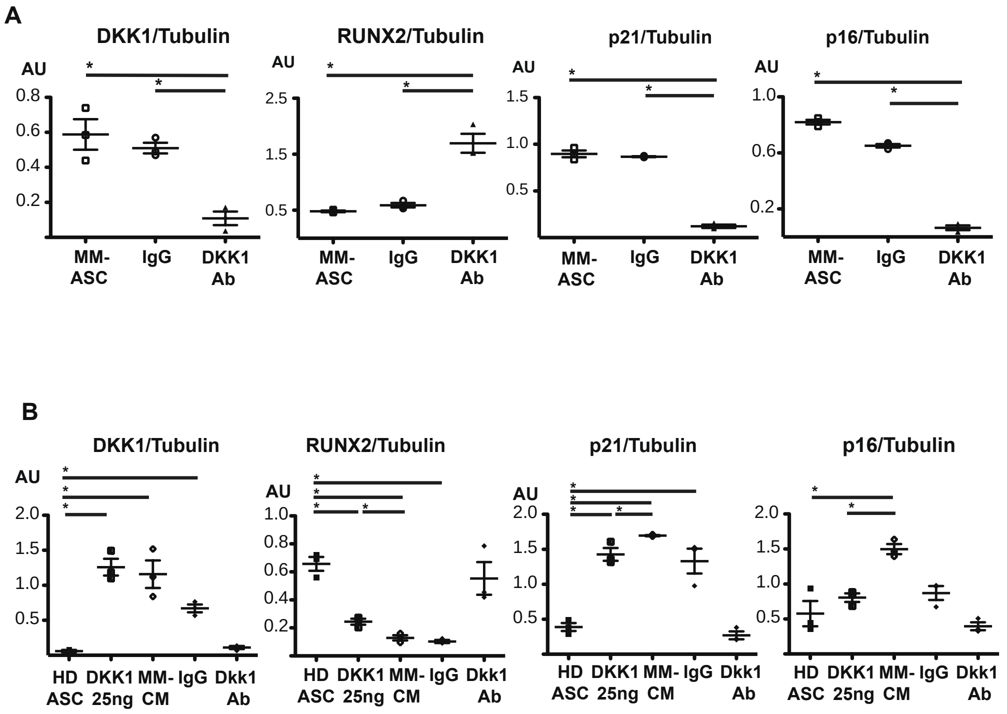

Figure Sup 3: DKK1 decreased expression improves osteoblastogenesis .A MM-ASC were cultured without or with an anti-DKK1 monoclonal antibody (DKK1-Ab) or IgG as control. Whole cell lysates were extracted and analyzed by immunoblotting. Western blot quantifications are shown. **A** The reverse experiment was performed by adding to HD-ASC cultures either recombinant DKK1 protein or conditioned medium (CM) from MM-ASC cultures, incubated or not with anti-DKK1. Western blot quantifications are shown. * $p < 0.05$.
